# Synthesis of carbon nanotubes with and without catalyst particles

**DOI:** 10.1186/1556-276X-6-303

**Published:** 2011-04-07

**Authors:** Mark Hermann Rümmeli, Alicja Bachmatiuk, Felix Börrnert, Franziska Schäffel, Imad Ibrahim, Krzysztof Cendrowski, Grazyna Simha-Martynkova, Daniela Plachá, Ewa Borowiak-Palen, Gianaurelio Cuniberti, Bernd Büchner

**Affiliations:** 1 IFW Dresden, P.O. Box 270116, 01069 Dresden, Germany; 2 Technische Universität Dresden, 01062 Dresden, Germany; 3 University of Oxford, Parks Road, Oxford, OX1 3PH, UK; 4 West Pomeranian University of Technology, ul. Pulaskiego 10, 70-322 Szczecin, Poland; 5 Nanotechnology Center, VSB Technical University of Ostrava, 17. listopadu 15, 70833 Ostrava-Poruba, Czech Republic; 6 National Center for Nanomaterials Technology, POSTECH, Pohang 790-784, Republic of Korea

## Abstract

The initial development of carbon nanotube synthesis revolved heavily around the use of 3*d *valence transition metals such as Fe, Ni, and Co. More recently, noble metals (e.g. Au) and poor metals (e.g. In, Pb) have been shown to also yield carbon nanotubes. In addition, various ceramics and semiconductors can serve as catalytic particles suitable for tube formation and in some cases hybrid metal/metal oxide systems are possible. All-carbon systems for carbon nanotube growth without any catalytic particles have also been demonstrated. These different growth systems are briefly examined in this article and serve to highlight the breadth of avenues available for carbon nanotube synthesis.

## Introduction

**Figure 1:**
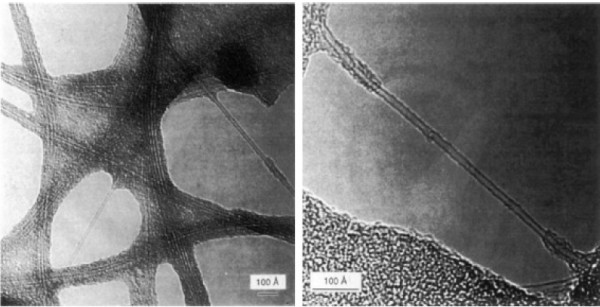
**Transmission electron micrographs of SWNT bundles (left panel) and an individual SWNT (right panel) synthesized from cobalt by Bethune et al**. Reprinted with permission from Bethune et al. [5].

The current excitement in carbon nanotubes (CNTs) was triggered by Sumio Iijima's Nature publication in 1991 [1]. At that time there was a considerable interest in developing the arc evaporation method, initially discovered by Huffman and Krätschmer [2], for the production of C_60 _in macroscopic amounts. Iijima analysed the deposit on the cathode and found macroscopic amounts of multi-walled carbon nanotubes (MWNTs) and facetted graphitic particles. The lack of fullerenes in the sample was unexpected. Moreover, the excitement at that time in carbon nanostructures, born out of the discovery of fullerenes [3] was a further favourable factor and so his publication drew significant attention. Iijima's next step was to see if he could fill these structures with transition metals. Transition metals were mixed into the graphitic electrodes and the arc evaporation process was run. The resultant product sprung another surprise. This time, a new form of carbon nanotube, namely, single-walled carbon nanotubes (SWNTs) with diameters between 1.1 and 1.3 nm were obtained [4]. Almost at the exact same time Donald S. Bethune, at IBM research laboratory, made the same discovery (see Figure 1) [5]. The discovery of SWNT was particularly exciting due to interesting structure-property correlations. In addition, it highlighted the use of transition metals as catalysts for carbon nanotube synthesis. Over the next years, a massive amount of synthesis routes and variations were developed. Most of these were based on the use of catalyst particles, including the chemical vapour deposition (CVD) route. CVD synthesis of CNT is facile and can be set up in laboratories without difficulty. Moreover, it is easily scaled up for mass production and so has developed into the most popular technique.

## Metal catalyst particles

**Figure 2:**
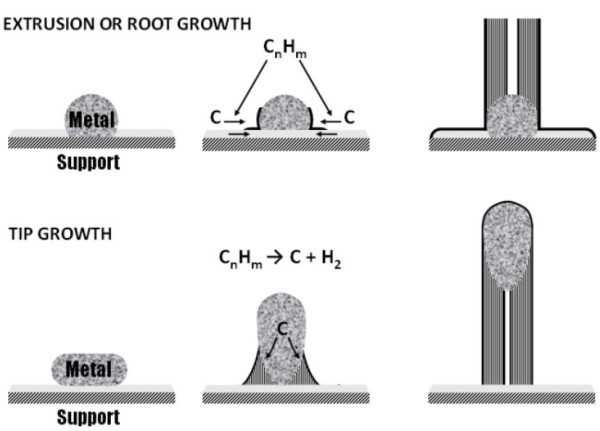
**Schematic showing base growth and tip growth of carbon fibres according to the VLS mode described by Baker **[13].

Vapor-grown CNT generally use metal catalyst particles and some even claim CNT synthesis requires a catalyst for their formation, despite Iijima's original work on MWNT synthesis never having used a catalyst. The use of metal catalysts and filamentous carbon from vapour-based routes has a long history dating back well before Iijima's landmark work, perhaps even as far back as 1889 [6]. For the most part 3*d *valence transition metals such as Fe, Co and Ni were used for the catalytic growth of CNT. More recently, several groups have grown CNTs from metals such as Au, Ag and Cu [7-10] and poor metals, e.g. Pb, In [11,12]. The conventional arguments for CNT growth are argued to occur in a similar manner to the model proposed for filamentous carbon growth by Baker et al. [13] (Figure 2) which is derived from the vapour-liquid-solid (VLS) theory developed by Wagner and Ellis to describe Si whisker formation [14]. The model proposed that hydrocarbons adsorb on the metal particles and are catalytically decomposed. This results in carbon dissolving into the particle forming a liquid eutectic. Upon supersaturation, carbon precipitates in a tubular, crystalline form. However, various alternative models exist and it is likely that the appropriate description of growth depends on the synthesis route and conditions used. For example, it is argued that at low temperature CNT growth can occur through surface diffusion [15]. In addition, most models assume thermal equilibrium conditions, although in practice, this is not so. In the case of noble metal catalyst particles, at temperatures where the VLS model is expected to be valid, they exhibit very low carbon solubility and negligible carbide formation. Zhou et al. [16] argue that low carbon solubility results in an increased precipitation rate. To grow carbon nanotubes, Lu and Liu [17] argue one needs to match the carbon supply rate to the tube formation rate.

## Ceramic and semiconductor catalysts

**Figure 3:**
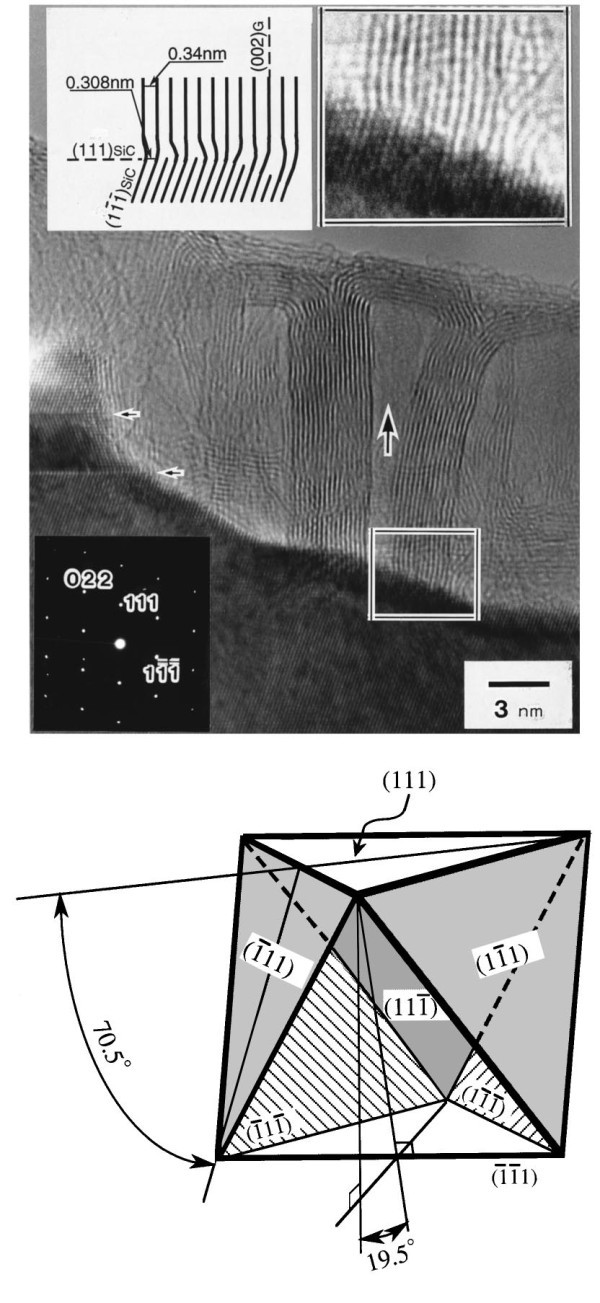
**Transmission electron micrograph of the interface between the graphite constructing a carbon nanotube and β-SiC on the surface of (111) β-SiC**. Lower panel: Schematic of the orientation relationship between one [111] SiC plane, on which carbon nanotubes are standing perpendicularly, and the other [111] SiC planes. Reprinted with permission from Kusunoki et al. [18].

Of the non-metallic catalysts for CNT, SiC is the most widely used and historically one of the first to be exploited. The early investigations involved the high temperature annealing (>1500°C) of SiC and was first demonstrated by Kusunoki et al. [18]. An example of the CNT is provided in Figure 3. Kusunoki and co-workers showed that in low vacuum conditions the SiC decomposes through the following oxidation route:(1)

The controlled oxidation process depletes Si at the surface, enabling the construction of CNTs. However, the formation of the initial caps at the nucleation stage has yet to be clarified [19]. Some argue a transformation process of surface graphene layers [20,21] or amorphous carbon [22] forms nucleation caps. Others argue the formation of convex structures on the surface enable initial cap formation [23-25]. Single-walled carbon nanotubes (SWNTs) can also be grown from SiC nanoparticles in CVD as was shown by Takagi [26]. Botti et al. [27,28] demonstrated laser annealing of SiC nanoparticles as a technique to obtain CNT.

The potential of semiconducting catalyst particles was first demonstrated by Uchino et al. [29,30] in which carbon-doped SiGe islands on Si were used to grow CNT after chemical oxidation and annealing treatments. Growth of the CNT was argued to occur from Ge clusters.

This is due to the greater thermodynamic tendency of Si to be oxidized as compared to Ge. Thus, the oxidation treatment results in the formation of SiO_2 _and the segregation of Ge clusters. Takagi et al. [26] also showed that SWNT could be grown directly from Ge particles as well as from Si nanoparticles.

**Figure 4:**
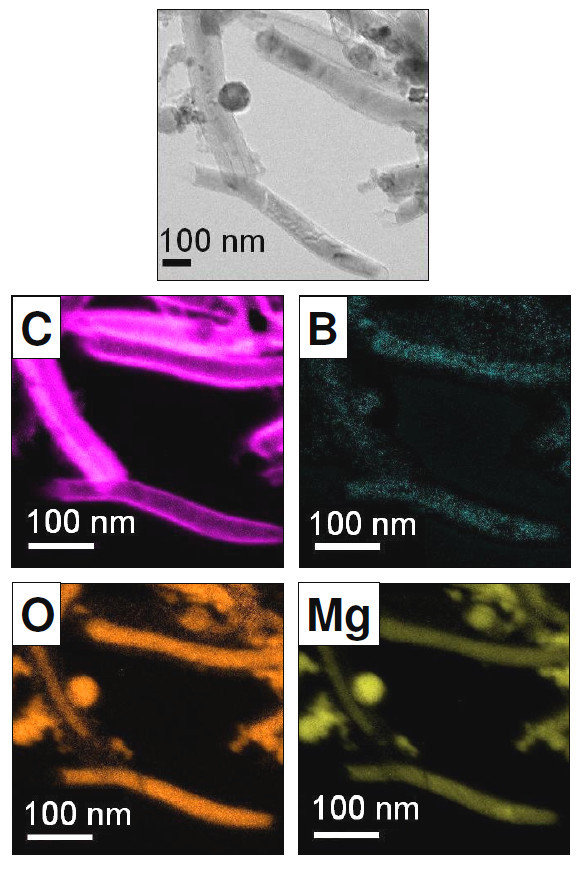
**Energy filtered TEM images of carbon nanotubes produced from phenylboronic acid in a MgO matrix**. The images show a carbon outer shell and a core (nanowire) comprised B, O and Mg. Top image-zero loss image. The C, B, O and MgO energy filtered TEM images are presented in false colour. Reprinted with kind permission from Bachmatiuk et al. [34].

Numerous investigators have shown oxides are well suited for CNT growth. An early example was the use of MgO as the catalysts for SWNT formation via the laser evaporation route [11]. More recently, Liu et al. [31] showed Al_2_O_3 _nanoparticles could be used to grow SWNT using an alcohol CVD route. Steiner et al. [32] showed both multi- and single-walled carbon nanotubes could be grown from zirconia. The use of magnesium borates can yield B-doped CNT (Figure 4) as was first demonstrated by Bystrzejewski et al. [33,34].

In 2009, two groups showed SWNT formation using SiO_2 _nanoparticles [35,36]. A little later Bachmatiuk et al. [37,38] showed stacked cup CNT could be grown from amorphous SiO_2 _nano-particles. However, transmission electron microscopy (TEM), infrared (IR) and Raman spectroscopic studies showed the nano-particles at the root of the CNT to be SiC. Their data points to the carbo-thermal reduction of SiO_2_. This result is in contrast to X-ray photoemission studies (XPS) by Huang et al. [36] which did not show any carbide formation and hence they argued growth occurred from the SiO_2 _particles. Steiner et al. [32] also conducted XPS studies and also found no evidence for carbide formation when using zirconia as the catalyst. However, it should be noted that Bachmatiuk et al. [37] also found no carbide formation when using XPS despite other techniques clearly demonstrating the presence of carbides. This suggests XPS, which is a surface sensitive technique, may not be best suited to determine if oxides used as catalysts for CNT growth reduce to carbides or not during synthesis. Various other oxides, outside of those mentioned, including TiO_2 _and lanthanide oxides can also be used to grow carbon nanotubes [36]. Templated CNT grown in porous alumina without catalyst particles have also been demonstrated [39]. Further studies are required to better understand which oxide systems are stable and which are reducible. Previous studies of ours in which nano-crystalline oxides were subjected CVD reactions showed many oxides are stable, whilst others are not. These studies confirmed oxides are capable of graphitising carbon [40].

## Hybrid metal/metal-oxide catalyst systems

Many of the oxides described above as catalytic nano-particles for CNT growth are often used as supports in supported catalyst CVD. Commonly used oxide supports are Al_2_O_3_, SiO_2_, TiO_2 _and MgO. All these oxides have been shown to grow CNT. Their role is primarily to stabilize the metal catalysts, viz. prevent coalescence. However, in oxide-supported metal catalysis it is well known that small clusters can have enhanced catalytic activity. A well-known example is Au, which is a bulk material is rather inert, but finely dispersed and deposited on oxides as small nano-clusters Au exhibits high catalytic ability (e.g. Haruta[41]). This enhanced catalytic activity is generally accepted to occur at the circumference of the nano-cluster/support interface.

**Figure 5:**
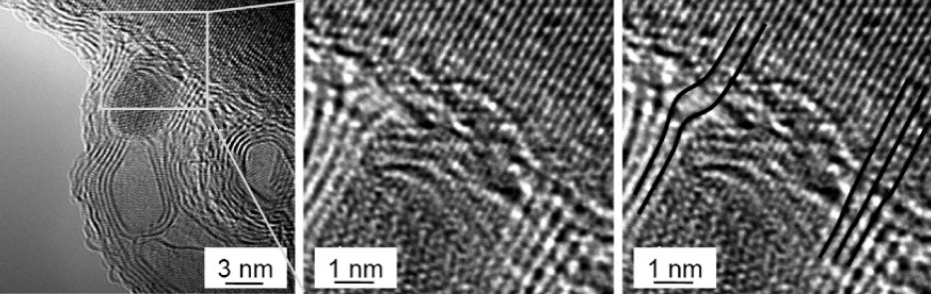
**TEM micrographs showing cross section view of a CNT root at the support surface**. The (Co) catalyst particle resides in the core of the tube. The fringes at the base of the particle correspond to the (200) lattice fringes of cubic Co. The outer walls of the CNT align themselves with the lattice fringes of the α-alumina nanoplatelet. The middle micrograph is a magnification of the boxed region from the left micrograph. The right micrograph is a copy of the middle image with lines added to highlight the alignment of the graphitic planes with the rhombohedral (110) lattice fringes of the corundum support. Reprinted from Rümmeli et al. [43].

It is then natural to query if oxides and the catalyst/support interface play a role in the case of CNT grown from oxide-supported metal catalyst clusters. To this end, we conducted various studies on CNT grown from Fe and Co clusters supported on alumina. Whilst the studies showed a good correlation between the initial catalyst size and the CNT outer diameter, after synthesis the catalyst particles are found to lie within the core of the CNT and are elongated [42]. In addition, the roots of the graphitic walls do not terminate on the metal particle but rather on the oxide support as shown in Figure 5[43]. This highlights the diversity with which carbon nanotubes can grow, in that some base growth modes show the CNT is rooted at the metal catalyst particle [44] much like tip growth grown CNT [45] or in other cases from the oxide support [42,43].

Another hybrid metal/metal-oxide example is the hydrocarbon dissociation over supported less active metal catalysts like Au and Cu, where it is argued that electron donation to the support creates *d*-vacancies for hydrocarbon dissociation [46].

## All carbon systems

**Figure 6:**
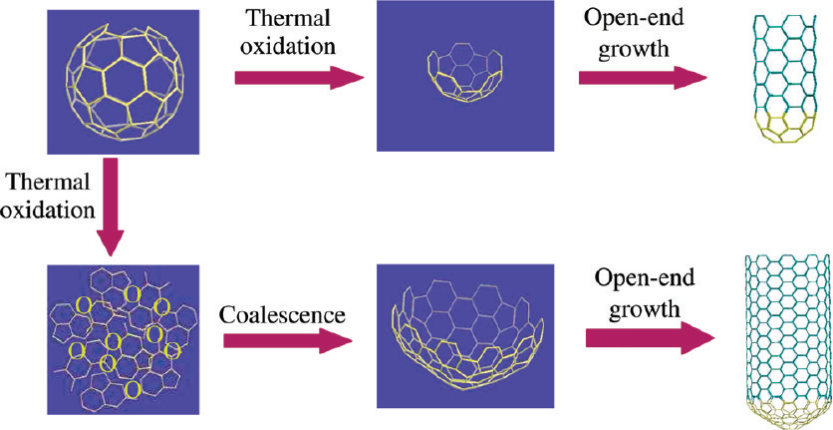
**Proposed mechanism for the growth of single walled carbon nanotubes using thermally opened C_60 _caps according to Yu et al**. [50]. Reprinted with permission.

The formation of CNT on the cathode in the arc-discharge route can occur without catalyst addition as shown by the work of Bacon in 1957 [47] and more recently by Iijima [1]. Despite the huge impact of Iijima's 1991 Nature paper, the fact that no catalyst was required was largely ignored or forgotten. More recently, a broad array of growth routes using pure carbon systems without any catalyst particle addition have emerged. Takagi et al. [48] have shown that SWNT can be grown in CVD using nano-diamond particles as catalysts. Moreover, nano-diamond particles do not suffer from coalescence and sintering difficulties. Exciting strategies to open fullerenes and use them as nucleation caps for SWNT have also been demonstrated. Once the fullerenes have been opened they are subjected to a CVD process and grow tubes [49,50]. The proposed growth mechanism is given in Figure 6. In a similar vein, the direct cloning of SWNT was shown by Liu and co-workers [51]. The formation of CNT on graphitic surfaces has also been demonstrated in various works by Lin et al. [52,53]. In these studies by Lin et al., it was shown that the early formation of amorphous nanohumps apparently serve as seed sites for the self-assembly of CNT.

## Growth Mechanisms

**Figure 7:**
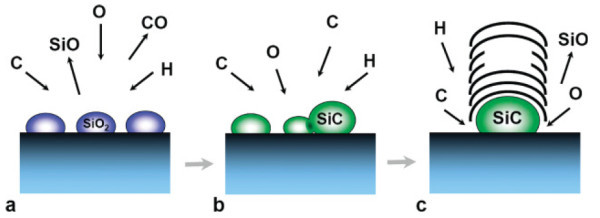
**Schematic representation of the carbothermal reduction of silica to silicon carbide and carbon nanostructure formation**: **(a) **SiO_2 _is reduced to SiC via a carbothermal reaction, **(b) **SiC nanoparticles coalesce, **(c) **carbon caps form on the surface of the SiC particles through precipitation and/or SiC decomposition. Reproduced with permission from Bachmatiuk et al. [37].

Whilst significant strides have been made in understanding CNT synthesis, the mechanisms behind growth remain a highly debated issue. In part this is due to some mechanisms being presented as universal. The brief variety of synthesis strategies presented in this simple review alone, highlight the need for particular mechanisms for specific routes and conditions. It is generally accepted that VLS description presented by Baker et al. [13] for carbon filament growth is also applicable to carbon nanotube growth, at least when metal catalyst particles are employed. However, even in this case, there are inconsistencies. As Reilly and Whitten [54] pointed out, the so called catalyst poisoning has yet to be demonstrated. As they highlight, often it is argued that a metal catalyst particle coated with amorphous carbon is considered poisoned, yet when it is coated with graphitic carbon (CNT growth) it is not considered poisoned, viz. they are apparently still able to decompose hydrocarbons. This oddity is further illustrated by our studies in which the catalyst particles lie fully within the core of the CNT [42,43]. Moreover, the ability of oxides to form graphene [40,55] and CNT [26-38] with out any metal catalyst present further weakens the commonly accepted notion that the (metal) catalyst particle is required to decompose the hydrocarbon. Reilly and Whitten proposed a free radical condensate (FRC) forms which provides carbon species through a leaving group. The breaking of carbon-hydrogen or carbon-carbon bonds naturally form free-radicals in hydrocarbon pyrolysis, with each fragment keeping one electron to form two radicals. The presence of a radical in a hydrocarbon molecule enables rapid rearrangement of carbon bonds. This same argument can explain the nucleation of CNT from unstable nano-humps which form on graphitic surfaces which then eventually lead to the formation of multi-walled carbon nanotubes [52,53]. Thus, in the FRC model, the catalyst particle's primary role is to serve as template for the formation of hemispherical caps at nucleation (as this reduces the high total surface energy of the particle caused by its high curvature). Thereafter, the catalyst may also provide an interface where carbon rearrangement may occur. However, this is not a prerequisite. Another surface, for example, an oxide support or simply unsaturated bonds at the edges of graphitic layers (e.g. open tube ends) can provide suitable sites for growth. Various studies provide experimental evidence for carbon addition to the edges of free standing graphitic edges [56-58]. In this scenario, carbon species are able to diffuse along the surface of graphitic layers which are then adsorbed at the edges. This self-assembling mechanism can explain the growth of cloned SWNT [51], SWNT nucleated from opened fullerenes [49,50] and from MWNT grown on graphitic surfaces [52,53]. In the case of CNT growth from stable oxides (oxides which are not reduced in the reaction), either in nano-particulate form or as the support material, the VLS theory is not valid since carbon dissolution is unlikely and probably occurs through surface diffusion processes. In the case of very small (<5 nm) non-metallic catalyst particles, the increased relative fraction of low-coordinated atoms could lead to surface saturation followed by carbon precipitation [7]. On the other hand, where the oxide can be reduced to a carbide, as for example, the carbo-thermal reduction of SiO_2 _nanoparticles [37,38], bulk carbon dissolution and precipitation in a manner similar to the VLS theory may be relevant (e.g. Figure 7).

In short, there appear to be a variety of growth modes and investigating each is complicated. Ex situ studies by definition means the catalysts have had time to relax and re-crystallize before being subjected to any investigative method. Hence, ex situ studies are necessarily limited in that they cannot unequivocally testify to circumstances during growth. On the back of this some argue in situ measurements as the only way forward. However, these routes present key limitations such as the need to work at very low pressures, well beyond any conventional or commercial route would use, as is the case for TEM and XPS in situ studies. Moreover, in in situ TEM only tiny sample sizes are examined and in the case of XPS in situ examinations, as already discussed above, the technique is surface sensitive and hence provides limited information on the catalyst during growth. Another area to investigate is how nature produces carbon nanotubes. Surprisingly, there is little evidence on planet Earth for their formation with only a few examples of MWNT and none for SWNT [59]. However, CNT may form more readily in outer space. Graphite whiskers have been found in high-temperature components of meteorites [60]. In addition, it has been proposed they can form in protostellar nebulae via Fischer-Tropsch-type catalytic reactions [61,62]. Recent experiments by the same group investigating the potential of Fischer-Tropsch and Haber-Bosch type reactions appear to support this hypothesis [63]. Thus, it is the collective data from both ex situ and in situ examinations that are important; however, the limitations of each implemented technique, and the specifics of the synthesis route in question must be considered as there is no single universal growth mode.

## Summary

There remains a fair amount of controversy in explaining carbon nanotube growth; this in part is due to the sheer number of possible synthesis routes and the fact that there is no single universal growth mode. Even so, tremendous advances have been made. This includes the development of new catalyst systems and even catalyst-free systems. Nonetheless the successful integration of CNT into applications and large-scale production processes remains limited and is dependant on the understanding of several fundamental issues. Some of these issues are highlighted by the disparate catalyst and catalyst free options available which raise new questions on nucleation and growth as well as the role of supports in supported catalysts. In some sense the rapid development of graphene may render CNT less important, for example, in the integration of carbon nanotubes in integrated circuit manufacturing, however, many of the questions raised in understanding carbon nanotube growth are directly relevant to graphene also.

## Abbreviations

CNT: carbon nanotubes; CVD: chemical vapour deposition; FRC: free radical condensate; IR: infrared; MWNTs: multi-walled carbon nanotubes; SWNTs: single-walled carbon nanotubes; TEM: transmission electron microscopy; VLS: vapour-liquid-solid; XPS: X-ray photoemission studies.

## Competing interests

The authors declare that they have no competing interests.

## Authors' contributions

MHR designed the manuscript layout. MHR, AB, FB, FS, II, KC, GS-M, DP, EB-P, GC and BB participated in some of the studies and participated in the drafting of the manuscript. All authors read and approved the final manuscript.
